# The Marine Fish Gut Microbiome as a Source of Novel Bacteriocins

**DOI:** 10.3390/microorganisms12071346

**Published:** 2024-07-01

**Authors:** Shona Uniacke-Lowe, Catherine Stanton, Colin Hill, R. Paul Ross

**Affiliations:** 1 Teagasc Food Research Centre, Moorepark, P61 C996 Fermoy Cork, Ireland; 2 APC Microbiome Ireland, Cork, Ireland; 3 School of Microbiology, University College Cork, T12 K8AF Cork, Ireland

**Keywords:** marine fish, gut microbiome, bacteriocins, bioprospecting, antimicrobial

## Abstract

The marine environment is the largest ecological habitat on Earth, albeit one of the least explored, particularly in terms of its microbial inhabitants. The marine fish gut is host to a diverse microbial community from which diverse bioactive molecules can be sourced. Due to the unique environmental pressures these microbial communities experience, the bioactive molecules they produce often evolve unique adaptations that give them diverse structures and activities, differentiating them from terrestrial homologues. Of particular interest, due to their structural and functional diversity, are the ribosomally-synthesized antimicrobial peptides (bacteriocins). With increasing pressure from emerging antibiotic-resistant disease and industrial demand for novel therapeutics, the marine fish gut microbiome represents a relatively untapped resource of novel bacteriocins that could prove beneficial to human health and aquaculture. This review presents an overview of the marine fish gut microbiome and explores its potential as a source of bacteriocins for human health with considerations for applications and future research in this area.

## 1 Introduction

The marine environment is the largest ecological habitat on the planet. The Earth’s oceans cover over 70% of the planet’s surface and reach depths of over 10,000 m [1]. Each layer, or zone, of the ocean is its own ecological niche that is distinguishable based on factors such as temperature, salinity and sunlight availability [2]. The top layers (epipelagic and mesopelagic zones) sustain a high abundance of diverse life and are very metabolically active. In contrast, the deep ocean layers (bathypelagic, abyssal and hadal zones) are subject to low temperatures, high hydrostatic pressure and lack of sunlight [3]. From hydrothermal vents to the deep, dark trenches, marine fish inhabit extreme environments and have as a result acquired diverse and unique physiological and molecular adaptations.

The marine environment is also a rich source of diverse natural bioactive compounds with therapeutic and economic potential. To date, there are 15 clinically approved drugs derived from marine organisms, ranging from peptides to antibodies and covering a wide variety of biological activities. Many more are in various stages of clinical trials [4]. We are in an age when there is a huge demand for novel antimicrobials and alternative treatments to antibiotics. Antimicrobial resistance (AMR) is an extremely concerning and global threat to human health. It has been estimated that in 2019 alone, AMR was associated with 4.95 million deaths [5]. Traditional antibiotic treatments have become less effective, multidrug-resistant pathogens are more common yet the development of new antibiotic-based treatments has slowed dramatically over the past few decades [6]. From 2014 to 2018 over 250 preclinical antimicrobial compounds from marine organisms were reported [7–9]. The majority of these compounds were polyketides (35%), terpenoids (26%), alkaloids (17%) and peptides (14%), as well as lipids, lipopeptides and several other molecule types. Just over half of these molecules were produced by bacteria or fungi, and, significantly, these producers were isolated from higher organisms, particularly from marine sponges. Yet none of these preclinical marine-derived antimicrobials are bacteriocins.

The mammalian gut microbiome is a well-established source of beneficial bacteria and compounds with therapeutic potential [10,11], and undoubtedly, this scenario is paralleled in the fish gut microbiome. There is growing interest in bioprospecting the fish gut micro-biome to find novel bioactive compounds with therapeutic potential and for applications in aquaculture [12]. The gut microbiome of fish has crucial biological functions in digestion, immune system modulation, stress response, and protection from pathogens/infection [13]. Advances in our understanding of the specific functional roles of the marine fish gut microbiome has been recently reviewed by Ou et al. [14]. For example, it has been shown that microorganisms of the marine fish gut protect the host from infection by pathogenic species through the production of various metabolites and antimicrobial substances, such as bacteriocins.

Bacteriocins are low molecular weight (<10 kDa), ribosomally-synthesized peptides with antimicrobial activity produced by bacteria that are immune to their own bacteriocin. Bacteriocins are usually classified into two major classes, based mainly on the extent to which they are modified. Class I bacteriocins are those which undergo extensive post-translational modification (e.g., lantibiotics or lanthipeptides), and class II bacteriocins are unmodified or cyclic [15]. The class I lanthipeptides, for example, are synthesized as prepeptides with unique lanthionine and/or β-methyllanthionine residues that undergo extensive posttranslational modification resulting in the formation of signature (methyl)lanthionine rings. Lanthipeptides can be further divided into subclasses I–V based on the enzyme(s) involved in their biosynthesis, modification, and mode of action [16]. The class II bacteriocins are subdivided into four groups (classes IIa–d): the anti-listerial pediocin-like peptides (IIa), two-component peptides (IIb), cyclic/circular bacteriocins (IIc) and the linear and non-pediocin-like peptides (IId) [17].

**Figure 1:**
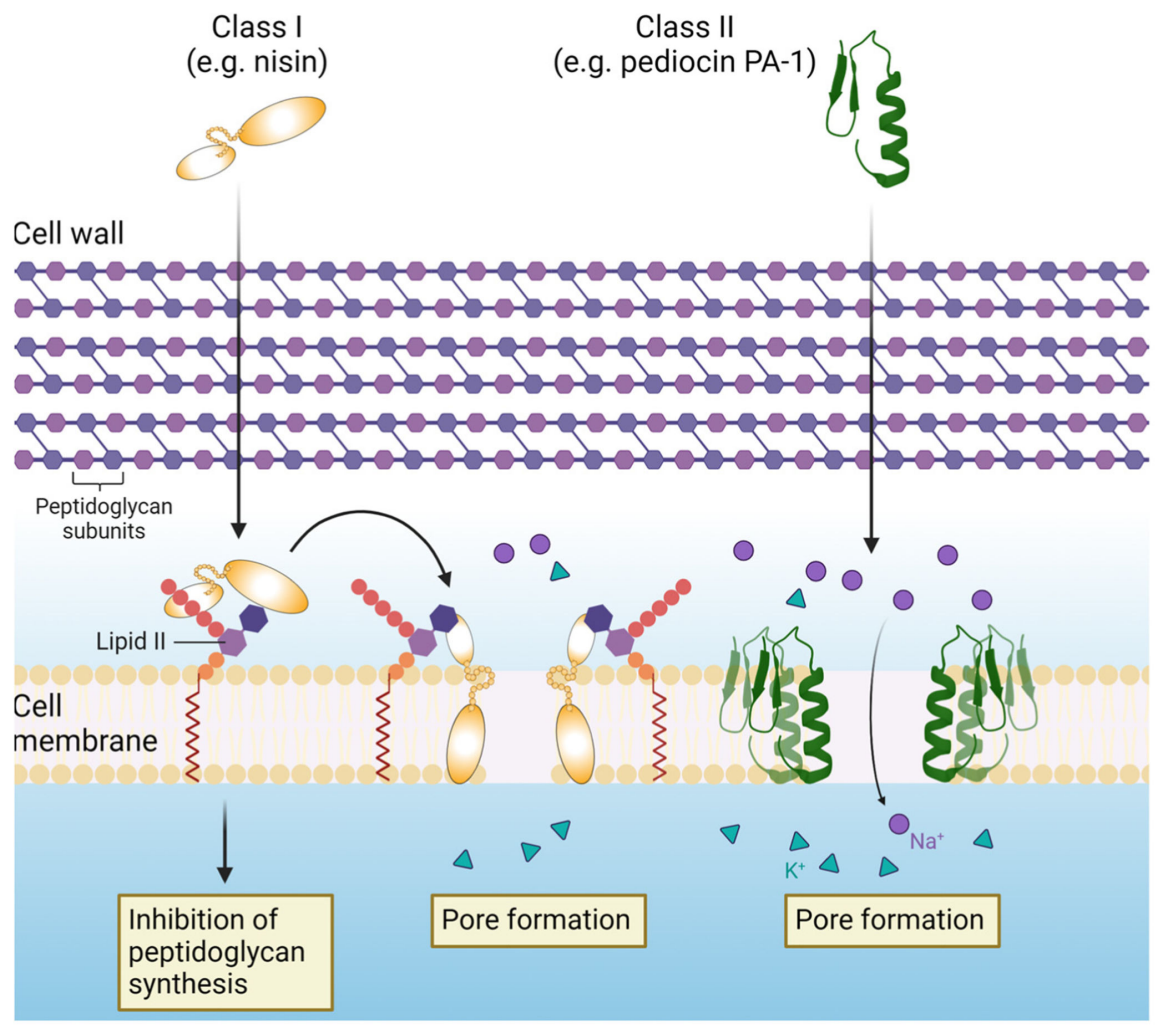
Representative illustration of the mechanism of action of bacteriocins. In general, bacteriocins form pores in target cell membranes, leading to leakage of intracellular contents and disruption of the membrane potential. They may also interfere with cellular processes by binding to specific receptors. For example, the class I bacteriocin nisin binds to lipid II, inhibiting peptidoglycan synthesis, and forms pores in the membrane.

Bacteriocins broadly target the cell membrane of susceptible cells (Figure 1). The hydrophobic nature and overall positive charge of bacteriocin molecules enables them to interfere with negatively-charged target cell membranes quite effectively. Though the different classes of bacteriocins have variable mechanisms of action, in general, they form pores in cell membranes leading to leakage of intracellular contents, disruption of the membrane potential and eventual cell death, or they can interfere with cellular processes by binding to specific cellular receptors [15,18]. The antimicrobial activity of bacteriocins can be described as either broad or narrow-spectrum. Broad-spectrum bacteriocins, as with conventional antibiotics, target a wide range of microbial species. This can be advantageous in treating infections wherein the causative pathogen is unknown. Consequently, however, this can cause significant disruption to commensal gut microbiota diversity and lead to an increased risk of developing chronic gut-associated diseases and antimicrobial-resistant strains [19]. Narrow-spectrum bacteriocins only target specific genera or even species. This characteristic can be utilized to develop precise agents for targeting pathogenic species without harming beneficial bacteria. For example, thuricin CD, a sactipeptide produced by *Bacillus thuringiensis*, was shown to effectively reduce *Clostridium difficile* in a distal colon model whilst having no significant effect on the overall microbial diversity, unlike the treatment with conventional antibiotics [19]. Bacteriocins are therefore emerging as promising alternatives to conventional antibiotics, not only due to their spectrum of antimicrobial activities, but also for their thermostability, and great capacity for bioengineering and generation of variants with value-added properties [15].

Attention is now turning to fish microbiomes as novel sources of bioactive molecules such as bacteriocins. Conventionally, the diversity of fish microbiomes has been determined using culture-dependent methods and has very likely been underestimated in terms of its abundance and richness. However, ongoing advances in culture-independent methodologies, such as metagenomic sequencing, are enabling more accurate analysis of this microbial niche. Because of the huge expanse of the marine environment, marine microorganisms are among the least explored and least accessible and thus the application of culture-independent metagenomic analysis becomes even more valuable. The marine fish gut microbiome is essentially an untapped reservoir for novel antimicrobials.

In this review, we discuss the diversity of the gut microbiomes of marine fish, with a focus on finfish: Chondrichthyes (cartilaginous fish) and Osteichthyes (bony fish). We also discuss current research on the discovery of bacteriocins from marine fish gut microbiota, their applications, and the current prospects of this microbial niche as a source of novel antimicrobials.

### 2 The Diversity of the Marine Fish Gut Microbiome

The marine fish gut is host to a diverse microbial community largely consisting of bacteria, with reported counts ranging from 10^4^–10^9^ colony forming units (CFU) [20], as well as fungi, archaea and viruses. This community begins to form at the fish larval stage, with early colonizers originating from the egg surface, the surrounding water and the first feed [21]. The diversity of the fish gut microbiome is then influenced by biotic and abiotic factors such as host phylogeny, trophic level (diet) and environmental salinity [22], as well as, though to a lesser extent, environmental pH and temperature [20,23]. The fish gut microbiome is an important and unique niche within the marine environment, with reports that the composition of microorganisms found in marine fish gut can differ from that of the surrounding waters and can even contain species that are rarely, if at all, found in the surrounding water [24].

The composition of the marine fish gut microbiome has already been extensively reviewed [14,20,25]. Early studies on fish microbiome diversity were limited to the culturable organisms [26,27], however, with the emergence of next-generation sequencing (NGS) and metagenomic technologies there is now a growing number of culture-independent studies that have been able to provide a more comprehensive description of the fish gut microbiome [28]. Findings from more recent studies which have utilized culture-independent methods are given in Table 1. The most frequently reported phylum is Pseudomonadota (formerly Proteobacteria), followed by Bacillota (Firmicutes), Actinomycetota (Actinobacteria) and Bacteroidota (Bacteroidetes). Other abundant phyla include Mycoplasmatota (Tenericutes), Cyanobacteriota (Cyanobacteria), Fusobacteriota (Fusobacteria) and Chloroflexota (Chloroflexi). At a lower taxonomic level, Vibrionales are frequently detected, particularly species of *Vibrio* and *Photobacterium*, as well as members of the families *Moraxellaceae, Pseudomonadaceae* and *Micrococcaceae* (Table 2). Host diet is a significant determinant of the predominant taxa detected within the gut microbiome. Results reported by Sullam et al. [22] suggested that Bacteroidales and Clostridiales dominate the gut microbiomes of herbivorous marine fish, whilst Vibrionales and Alteromonadales are dominant in carnivorous and omnivorous marine fish. Similarly, Huang et al. [29] demonstrated that host feeding habits could be differentiated by “indicators”, specific bacterial taxa. It was suggested that these indicators were associated with specific symbiotic functional activities such as the production of bioactive enzymes, thus aiding in digestion within the gut.

The majority of studies of marine fish gut microbiota have focused on 16S rRNA sequence data, profiling the bacterial portion of this community. However, one recent study analyzed whole-metagenome shotgun sequencing data from the intestines of various deep-sea fish of the northern Atlantic Ocean [30]. Overall, the most abundant phyla detected were Pseudomonadota, Bacillota, Actinomycetota and Bacteroidota, in agreement with previous studies. However, cluster analysis showed that the proportions of these phyla varied significantly between some samples. Furthermore, in one cluster a significant proportion of Eukaryota was detected, specifically Ascomycota, Basidiomycota and Euryarchaeota. In terms of the eukaryotic species, there have been suggestions that yeasts are commensals of the fish microbiome with species such as *Debaryomyces hansenii, Saccharomyces cerevisiae* and red-pigmented *Rhodotorula* dominating [25,31]. The main archaeal groups reported in studies of marine fish gut microbiota are Crenarchaeota and Euryarchaeota [32,33]. Euryarchaeota have also been identified in deep-sea fish microbiota samples [30]. These are two of the major groups of planktonic archaea found in marine environments and are key contributors to nutrient cycling in pelagic waters and deep-sea sediment [34]. It has been suggested that the digestive tract of fish is an important habitat for archaeal groups within the marine environment, particularly the obligate anaerobic Euryarchaeota [33].

Viruses are the most abundant entities on earth, and it is no different in the marine environment. It has been estimated that there are between 10^6^ and 10^8^ viruses per ml of seawater [35]. A recent study by Geoghegan et al. [36] used a metagenomic-based approach to characterize the viromes of several marine fish species: They identified viral sequences that represented 11 viral families, with *Astroviridae, Picornaviridae, Arenviridae, Reoviridae* and *Hepadraviridae* dominating. They suggested that many of the identified viruses were diet or microbiome -associated and that host phylogenetics is a significant determinant of virome diversity.

Efforts have been made to define a “core microbiome” for marine fish, however this has proven a difficult task given the diversity of marine fish and the factors that affect their microbiome diversity. Fish gut microbiota members can also be transient [20]. Instead, it has been suggested that, perhaps, functional diversity is more important than taxonomic/phylogenetic diversity [28,29]. Furthermore, many of these studies have noted that a high percentage of operational taxonomic units (OTUs) could not be taxonomically assigned at the genus level, indicating the novelty and high biodiscovery potential of the marine fish gut microbiomes. For example, Huang et al. [29] reported that over 70% of OTUs from coastal fish gastrointestinal samples were unassigned at genus level, whilst Johny et al. [37] reported over 90% of the deep-sea fish gut OTUs could not be assigned at genus level.

The gut microbiome of marine fish is involved in the regulation of host processes including digestion, stress and immune responses, reproduction, and metabolism [13,22]. Many of these processes are mediated by the production of microbial metabolites, including antimicrobials, and bioactive enzymes.

#### 3 Bacteriocins from Marine Fish Gut Microbiota

Bioactive molecules representing several different bacteriocin classes and bacteriocin-like peptides have been identified in isolates of marine fish gut microbiomes, as outlined in Table 3. These peptides exhibit activity against a wide range of gram-positive and gram-negative bacteria, and even against fungi in some cases. Though in many of these studies the chemical structure of these bacteriocins/peptides is yet to be determined, it again highlights the biodiscovery potential and novelty of this microbial niche. Notably, these bacteriocins are all from bacilli or lactic acid bacteria (LAB) strains, some of which are novel species or strains.

### 3.1 Bacteriocins from LAB

Lactic acid bacteria (LAB) are important members of the fish gut microbiome with cultivable species from marine fish including *Carnobacterium* spp., *Lactobacillus* spp., *Lactococcus* spp., *Enterococcus* spp. and *Pediococcus* spp. [54]. Studies have shown that beneficial LAB in the fish gut may have a significant role in promoting host fish health by improving resistance to infection through immune system modulation and the production of antimicrobial molecules, such as bacteriocins [54].

Pilet et al. [46] screened for antimicrobial-producing strains from the intestines of salmon and trout using *Listeria* species as target organisms. They identified two antimicrobial-producing species, *Carnobacterium piscicola* V1 and *Carnobacterium divergens* V41. *C. piscicola* V1 was found to produce two class IIa bacteriocins, piscicocins V1a and V1b. The purified peptides were active against the same spectrum of gram-positive indicator strains, which included *C. divergens, E. faecalis* and *L. monocytogenes*. Furthermore, piscicocin V1a was significantly more potent than V1b and was deemed to be a novel bacteriocin based on amino acid sequence analysis [47]. In a subsequent study, *C. divergens* V41 was found to produce divercin V41, also a novel class IIa bacteriocin. The amino acid sequence of divercin V41 was shown to be most similar to those of pediocin PA-1 and enterocin A [48]. Furthermore, piscicocins V1a and V1b and divercin V41 were optimally produced at temperatures below 20 °C and in the presence of up to 4% NaCl [55], likely an adaptation to the marine environment.

*Enterococcus mundtii* Tw56 was isolated from the intestine of an *Odontesthes platensis* specimen from the Patagonian region of Argentina, an area subject to relatively low temperatures. Cell-free supernatant (CFS) from this strain was able to inhibit gram-positive and gram-negative strains including *Enterococcus* spp., *Listeria* spp., *Pseudomonas aeruginosa* and *Shewanella putrefaciens*. It was deduced through thermal and chemical stability assays and PCR that the CFS contained mundticin KS, a class IIa bacteriocin [49].

**Figure 2:**
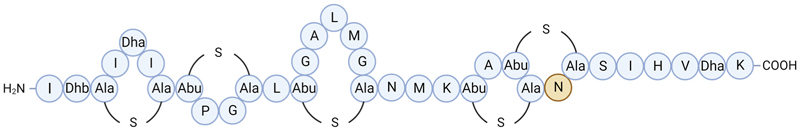
The structure of the class I bacteriocin (lanthipeptide) nisin Z, a natural nisin A variant, which differs by one amino acid (position 27 where Asn replaces Ala, highlighted in yellow). Nisin Z-producing *Lactococcus lactis* strains have been found in marine fish intestinal samples by two separate studies, Sequeiros et al. [45] and Heo et al. [44].

Two separate studies found nisin Z -producing *Lactococcus lactis* strains from marine fish intestines with activity against the fish pathogens, *Streptococcus iniae* and *Lactococcus garvieae*, respectively [44,45]. Nisin Z is a natural variant of the class I bacteriocin (lanthipeptide) nisin A, differing only by a single amino acid (His27Asn) [56] and contains the characteristic lanthionine and methyllanthionine rings of this bacteriocin class (Figure 2). As with other nisin peptides, nisin Z acts by binding to and forming pores in the membrane of target cells leading to cell death [57]. Sequeiros et al. [45] demonstrated this in their study when they exposed *L. garvieae* to supernatant from the nisin Z-producing fish isolate (*L. lactis* TW34) and reported a drop in viable cell counts by 6 logs (CFU/mL) within one hour but without cell lysis, thus indicating bactericidal activity. Heo et al. [44] prepared partially purified nisin Z in a solution containing varying concentrations of NaCl from 0–4% *w*/*v*. They showed that nisin Z prepared in 3.5% NaCl, a concentration similar to that of seawater, was the most effective against *S. iniae*. This demonstrates the increased effectiveness of the marine-derived nisin Z under native conditions.

More recently, Li et al. [51] isolated a novel class IId bacteriocin, CAMT6 from the marine fish isolate *Enterococcus durans* YQ-6. CFS from this strain possessed a broad spectrum of activity, targeting both gram-positive and gram-negative bacteria, and also inhibited fungi. Measurement of CFS conductivity and cell morphology imaging of *L. monocytogenes* after exposure to CAMT6 demonstrated that the mode of action of CAMT6 involved disruption of the cell membrane, causing leakage of cell contents. Furthermore, the purified CAMT6 peptide exhibited anti-*Listeria* activity in a chicken breast model and anti-biofilm activity, demonstrating a potential application in food preservation. The amino acid sequence of CAMT6 is unusual in that, at 12 amino acids in length, it is much shorter than previously reported bacteriocins from enterococci and did not share homology with bacterial-derived proteins, but, rather, was most similar to antimicrobial peptides from humans and sheep.

### 3.2 Bacteriocins from Bacilli

Bacilli isolated from marine fish have also been found to produce highly stable, and often novel, bacteriocins that can target a broad range of target strains:

Bindiya et al. (2015) screened isolates from the gut contents of a deep-sea shark (*Centroscyllium fabricii*) against various indicator strains. Amongst the antimicrobial producers, they identified *Bacillus amyloliquefaciens* BTSS-3, a strain with activity against pathogenic bacteria including *Salmonella enterica* Typhimurium, *Clostridium perfringens, S. aureus, Proteus vulgaris*, and several *Bacillus* species [52]. The purified antimicrobial molecule designated BaCf3, was subsequently shown to possess characteristics of being a bacteriocin: it had a mass of approximately 3000 Da, was pH tolerant (from pH 2.0–13.0) and highly thermostable (from 4–100 °C), retaining activity even after autoclaving [53]. Sequence compositional analysis found BaCf3 to be rich in hydrophobic amino acids, such as cysteine and glycine, and was predicted to contain at least one disulfide bridge. The predicted tertiary structure consisted of three anti-parallel β-sheets and resembled that of laterosporulin, a defensin-like bacteriocin from *Brevibacillus* sp. strain GI-9 [58,59]. Based on its hydrophobic nature and structural similarity to defensin-like bacteriocins, it was predicted that BaCf3 targeted the cell membrane. This was confirmed by microscopic analysis of *Bacillus circulans* target cells after exposure to BaCf3, whereby leakage of intracellular material and disruption to the cell membrane was observed [59]. Furthermore, BaCf3 was shown to also have anti-biofilm and anti-cancer activities [53,59]. This is one of the first studies that has purified a novel bacteriocin from a deep-sea fish gut isolate, and, significantly, it retained antimicrobial activity under low temperatures (4 °C) and after being subjected to high pressure and heat (autoclaving).

**Figure 3:**
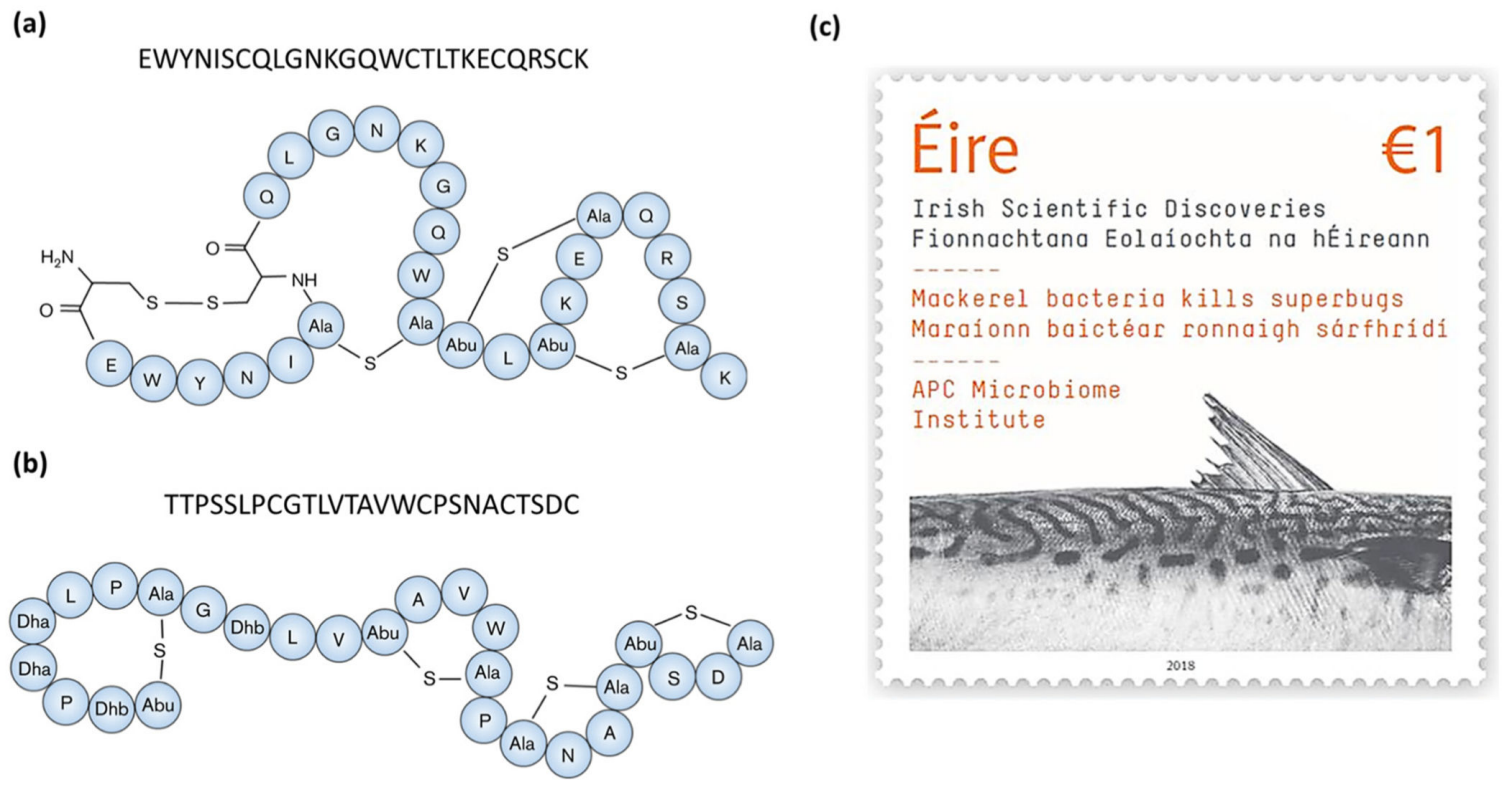
Predicted structures and core peptide sequences of the α (**a**) and β (**b**) peptides of Formicin, a novel two-component lanthipeptide produced by *Bacillus paralicheniformis* APC 1576, a mackerel gut isolate. Formicin’s secondary structure differs from that of other similar lanthipeptides, yet it still retains a broad spectrum of activity against gram-positive pathogens. Figure adapted from Collins et al. [43]. The discovery of formicin and its contribution to scientific research was commemorated by the release of a postal stamp released by An Post and Science Foundation Ireland, celebrating scientific discoveries in Ireland (**c**).

Formicin, a novel two-component lanthipeptide (class I bacteriocin), was discovered by screening bacterial isolates from the intestine of Atlantic mackerel (*Scomber scombrus*) for antimicrobial activity. The peptide, produced by a *Bacillus paralicheniformis* isolate, was found to inhibit a range of gram-positive indicator organisms including clinically relevant pathogens such as *Listeria monocytogenes, Staphylococcus aureus, Streptococcus mutans*, several species of *Enterococcus* and Clostridia, including *Clostridioides difficile* [43]. Formicin differs from other two-component lanthipeptides in that the α-peptide contains fewer hydrophobic amino acids and has an overall positive charge of +2, and the β-peptide uniquely contains a negatively charged C-terminus due to the presence of an aspartate residue in the penultimate position (Figure 3a, b). The discovery of formicin and its contribution to scientific discovery in Ireland was commemorated by the Irish national postal service (An Post) together with Science Foundation Ireland by the release of a special issue stamp in 2018 (Figure 3c).

Novel bacteriocin-like molecules derived from bacilli of the marine fish gut microbiome have also been reported, such as CAMT2 [60] and BpSl14 [61], produced by *Bacillus amyloliquefaciens* ZJHD-06 and *Bacillus safensis*, respectively. Both molecules exhibited a broad spectrum of activity, targeting both gram-negative and gram-positive strains and even fungi. Elucidation of their chemical structures found that they were more similar to eukaryotic AMPs than to bacterial peptides.

Recent studies combining in vitro and genomic screening have also highlighted the antimicrobial potential of novel Bacilli isolated from fish [62,63]. For example, *Bacillus* sp. GFP-2, a novel strain of *Bacillus velezensis*, was isolated from intestinal contents of whitespotted bamboo sharks (*Chiloscyllium plagiosum*) and found to inhibit the growth of *Bacillus subtilis* and *Escherichia coli*. Analysis of the genome sequence of strain GFP-2 found that it encoded genes for various AMPs, such as LCI and glyceraldehyde-3-phosphate dehydrogenase (GAPDH)-related peptides, various antimicrobial secondary metabolites including difficidin, bacillysin, macrolactin and fengycin, as well as lanthipeptides [64].

### 3.3 Bacteriocins from Actinobacteria

Actinobacteria are abundant in the marine environment, and those associated with higher organisms such as sponges, molluscs and fish are known to produce a wide variety of bioactive compounds with diverse biological activities [65]. One of the most significant antimicrobials from a marine-derived actinomycete is anthracimycin—an antibiotic with potent activity against *Bacillus anthracis*, the pathogen responsible for causing anthrax. Anthracimycin was discovered by screening *Streptomyces* isolates from marine sediment [66]. Indeed, *Streptomyces* are among the most frequently identified producers of bioactive compounds from marine environments, particularly those found in association with higher organisms [65]. Several recent studies have screened Actinobacteria from marine fish microbiomes for antimicrobial activity: Vignesh et al. [67] isolated Actinobacteria from marine fish gut samples that had antimicrobial activity against *S. enterica, S. aureus* and *E. coli*. Furthermore, one of the isolates, a *Streptomyces* strain, also demonstrated anti-fungal and quorum-sensing inhibitory activity. More recently, Vadivel et al. [68] isolated antimicrobial and anti-quorum sensing *Streptomyces* from marine fish gut samples. Several studies have highlighted that the key to identifying novel bioactive and antimicrobial compounds may now be in the “rare” Actinobacteria—those that are not as easily cultivated. These include genera such as *Salinispora, Actinomadura, Microbacterium* and *Micrococcus* [65,69]. Sanchez et al. [70] identified various Actinobacteria, including *Rhodococcus, Microbacterium* and *Micromonospora* species (which would be considered part of the “rare” Actinobacteria group) from marine fish digestive tract samples and detected antimicrobial activity against pathogenic *Vibrio* spp., *B. subtilis, S. aureus* and *E. faecium*. Though these studies didn’t identify specific antimicrobial molecules, they highlight the biodiscovery and antimicrobial potential of marine fish gut-derived Actinobacteria.

## 4 Applications of Marine Fish-Derived Bacteriocins

One of the applications for marine-derived bacteriocins is as an alternative to antibiotics in the treatment of fish diseases within the aquaculture industry [71,72]. Fish and fish products are significant, globally traded commodities, with millions of tonnes consumed each year, either directly as food, as fishmeal or for fish oil. Outbreaks of disease in commercial fish significantly impact global supply, economics and human health [73].

Many studies have also explored the application of bacteriocins from marine fish microbiomes, or the producing strains, in biopreservation of food products, such as seafood products [49,55] and chicken [51]. Schelegueda et al. [49], for example, assessed the potential use of mundticin KS from *E. mundtii* Tw56 in food biopreservation. The bacteriocins exhibited antimicrobial activity against *Listeria innocua* over a range of pH (2.0–10.0), and after heat treatment (up to 121 °C *×* 15 min). Furthermore, CFS from the producing strain retained full activity after storage at *−*30 °C for 1 year, indicating its potential for use in frozen foods. Duffes et al. [55] studied the anti-*Listeria* activity of the bacteriocinogenic strains, *C. pisccola* V1 and *C. divergens* V41, in vacuum-packed cold smoked salmon over a period of up to 4 weeks. In co-culture assays at 8 °C, both strains reduced the viability of *L. monocytogenes* by 5–7-fold (log CFU/g) compared to *L. monocytogenes* alone. The inhibitory effects were even greater at 4 °C, with *C. piscola* V1, for example, reducing the counts of *L. monocytogenes* to less than 10 CFU/g after 4 weeks. Crude bacteriocin extracts from the *Carnobacterium* strains also demonstrated effective anti-*Listeria* activity, particularly at 4 °C whereby *L. monocytogenes* was below detectable levels after 1 week. Significantly, the crude extracts were more effective at inhibiting *Listeria* than nisin under these conditions.

Bacteriocinogenic isolates from marine fish are generating great interest in the aquaculture industry for use as probiotics. For example, Shastry et al. [50] demonstrated that Enterococcus lactis RS5 exhibited not only bacteriocin production but also resistance to bile salts, low pH (53% viability at pH 1.5) and protease digestion—key attributes of probiotic strains. Nguyen et al. [74] explored the probiotic potential of the nisin Z-producing *L. lactis* WFLU12, originally isolated from the gut of olive flounder [44]. They reported that fish on a diet supplemented with 10^9^ CFU/g of strain WFLU12, exhibited increased growth and were more resistant to infection by *Streptococcus parauberis*, compared to the control group.

There is also the potential for use as treatments for infection in humans; indeed, many of the bacteriocins listed in Table 3 are capable of targeting human pathogens. Formicin, for example, exhibited effective activity against several clinically relevant species including *C. difficile* [43]. This is particularly important in cases of antimicrobial resistance whereby treatment with conventional antibiotics has become less effective and novel alternatives are sought.

## 5 Challenges, Metagenomics and Future Prospects

Some of the challenges in the biodiscovery of bacteriocins from marine microbial communities include the cultivation of the producing bacteria and expression of the bacteriocin biosynthetic genes under in vitro conditions. Without the correct conditions, potentially novel marine microorganisms, and thus their metabolites, may be lost when attempting to culture in vitro [75]. Environmental pressure, temperature and dissolved oxygen concentration are just a few factors to be taken into account as they can all affect the diversity of isolated microorganisms and the metabolites they produce [76–79]. Often, modified or selective media is required and long incubation times of days, or even weeks, for antimicrobial production to occur. Sanchez et al. [70], for example, implemented the use of selective media for the isolation of bioactive marine Actinobacteria from fish. Vadivel et al. [68] also explored optimization of antimicrobial production in *Streptomyces maritimus* SQA4, an isolate from squid, using the “one factor at a time” method, whereby one factor or variable in the cultivation step is modified at a time. They observed that substituting carbon, nitrogen, and salt sources, as well as altering pH, had varying effects on antimicrobial activity. Furthermore, production of the bioactive metabolite was detected when the strain was cultivated on solid medium after two days but was not detected when in broth culture until after 10 days of incubation. The growth of some marine bacteria may also be dependent on the presence of specific signaling molecules found in their natural environment [80].

Culture-independent and metagenomics-based technologies, such as next-generation sequencing, metagenomic library construction, metabolomics and genome mining are more frequently being used to study microbial communities [81]. Such methods allow for a more comprehensive understanding of these microbial communities and remove the bias of sampling only culturable microbiota. Frequently used genome mining tools for identifying bacteriocins and other secondary metabolite BGCs include BAGEL and antiSMASH, both standalone and user-friendly programs [82,83]. In using BAGEL (version 3) Collins et al. [43] could identify the formicin biosynthetic operon, from which the masses of the core bacteriocin peptides could be predicted. This was a critical step in the purification and characterization of formicin, as the peptide masses found using colony mass spectrometry did not match any previously characterized bacteriocins. AntiSMASH has been used to great effect to identify microbial secondary metabolite BGCs, such as polyketides, bacteriocins and non-ribosomal peptide synthetases (NRPs), from diverse marine habits including sponges [84,85], sediment [86] and Antarctic fish [87].

Recent advancements in biodiscovery technologies have also seen the development of machine-learning -based tools for high-throughput identification and classification of ribosomally synthesized and post-translationally modified peptides (RiPPs), such as RODEO [88] and DeepRiPP [89]. Other available tools for genome-mining of RiPP BGCs and advancements in this area of research have already been extensively reviewed else-where [90,91].

**Figure 4:**
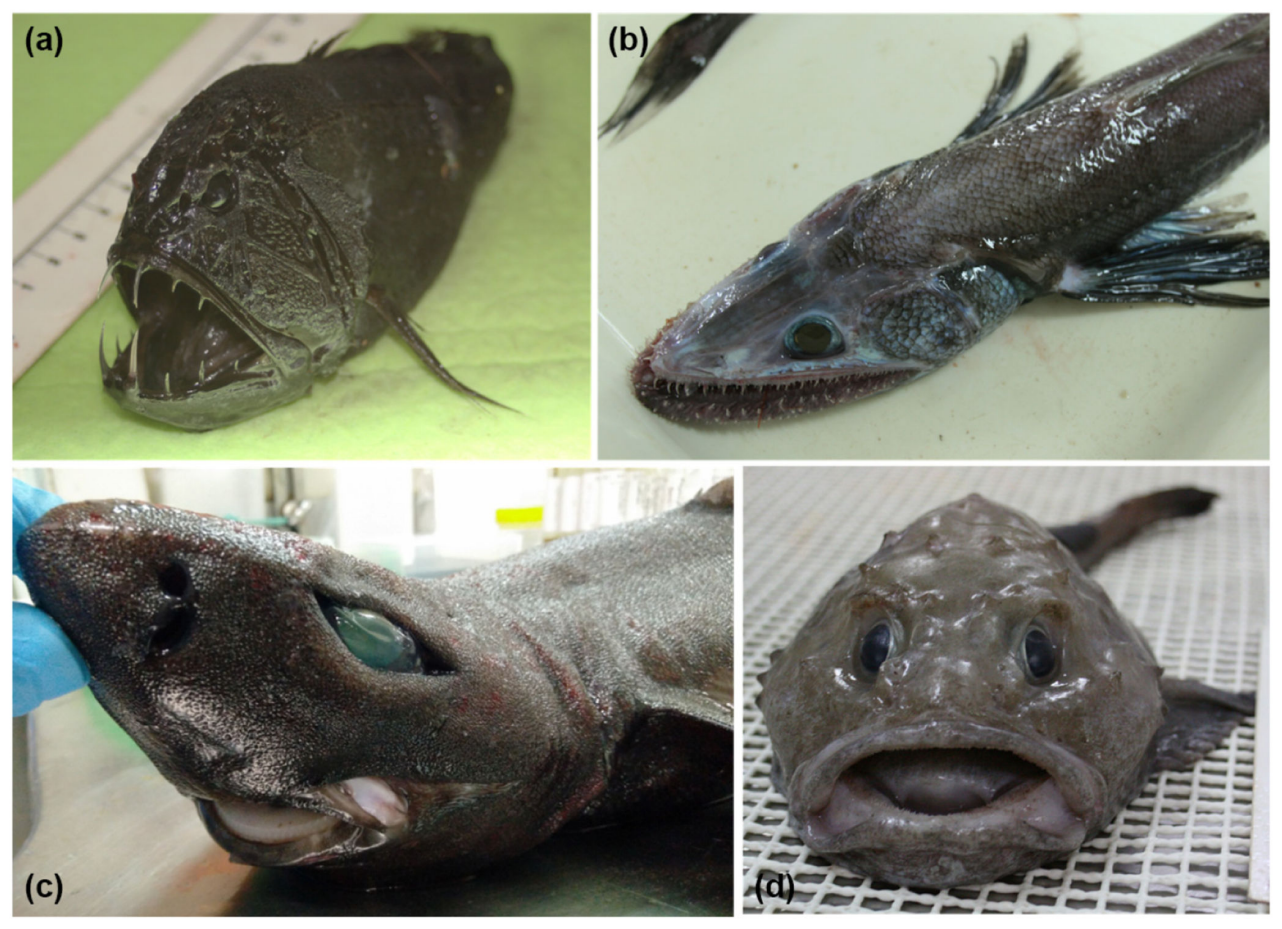
Culture-independent, metagenomics-based studies have enhanced our analysis of the microbiomes of marine fish, including rarer/unusual species such as *Anoplogaster cornuta* (**a**) *Bathysaurus ferox* (**b**) *Centroscyllium fabricii* (**c**) and *Cottunculus thomsonii* (**d**). Figure adapted from Collins et al. [93].

As mentioned above, culture-independent and metagenomic methods are being used to great effect in characterizing the taxonomic diversity of the marine fish gut microbiome. Several studies have also employed the use of metagenomic sequencing to characterize functionality, the presence of antimicrobial resistance genes and bioactive metabolite genes in marine fish microbiota [92–94], including those from rarer deep-sea fish species (Figure 4). The culture-independent nature of metagenomics allows for a more comprehensive understanding of these gut communities and removes the bias of sampling only culturable microbiota. Few studies, however, have used metagenomics approaches for screening marine fish microbiomes for bacteriocins specifically. Yi et al. [95] used metagenomic sequencing to study functional dynamics in the gut microbiota of several aquatic animals, including marine and freshwater fish. By comparative gene sequence analysis against multiple protein databases, they identified numerous bacteriocin-associated genes, including secretion systems, and immunity-related membrane transporter systems. However, they identified few core peptide biosynthetic genes, suggesting a role in antimicrobial resistance rather than production by the microbiota.

Whilst in silico genomics screening can identify bacteriocin biosynthetic gene clusters, there is still a need for peptide purification but expression in the native host can be challenging. In fact, it has been suggested that that majority of bacterial BGCs may be “silent”, in that the genes are identifiable yet there is no corresponding bioactivity detectable during in vitro experiments [96]. An emerging technology to combat this is bacteriocin “reincarnation”. This method involves cloning of the biosynthetic operon of an “inactive” antimicrobial into a heterologous host, thereby enabling controlled expression of the antimicrobial gene. Collins et al. [97] utilized this method to reincarnate pediocin-like bacteriocin structural genes that had been identified during in silico analysis of the *Lactobacillus* pangenome, yet these strains did not exhibit in vitro antimicrobial activity. Ten pediocin-like bacteriocin genes were cloned with a (pediocin) signal sequence and heterologously expressed in *E. coli* and *Lacticaseibacillus paracasei* and displayed antimicrobial activity against *L. innocua*. This study demonstrates the potential for such methods in the expression of such “inactive” bacteriocins, that may be identified in genomic sequences of marine-fish-derived bacteria, and allow for their heterologous expression under more familiar, terrestrial, conditions.

It may be that an approach using a combination of in vitro and in silico methods is key to the discovery of more novel bacteriocins from marine fish gut microbiota.

## 6 Conclusions

With the increasing demand for novel antimicrobial therapeutics to combat antimicrobial resistance, there is a pressing need to source novel bioactive compounds. The marine environment is vast, with a multitude of factors affecting the diversity of marine life and their biomolecules. The marine fish gut microbiome is no exception, and it is emerging as a productive source of antimicrobial molecules, such as bacteriocins. Key aspects of marine life, such as high salinity, hydrostatic pressure and a range of environmental temperatures all have an impact on the structural and functional diversity of marine antimicrobial molecules, including bacteriocins, often distinguishing them from terrestrial homologs. Characterizing the true diversity of microorganisms in marine fish gut microbiomes and the bioactive molecules they produce still presents challenges but may be key in determining the true potential of this niche. Recent applications of culture-independent technologies, such as metagenomics and next-generation sequencing, have begun to bridge this knowledge gap and are already being used to great effect to resolve the taxonomic diversity of the fish gut microbiome. Bacteriocins from the marine fish gut can be used to combat disease, either directly or indirectly as products of probiotic strains, and for the biopreservation of food products. The marine fish gut microbiome is a potential treasure trove of novel bacteriocins and other antimicrobials but limitations in accessibility and technology have meant its true bioprospective potential has yet to be truly explored.

## Figures and Tables

**Table 1:** The predominant phyla reported in recent culture-independent studies of marine fish gut microbiota. (phyla are listed in order of abundance where possible).

Fish Species	Sample	Predominant Phyla	Ref.
*Gadus morhua* (Atlantic Cod)	Intestinal contents	Pseudomonadota, Bacteroidota, Bacillota	[38]
*Siganus fuscescens* (Mottled spinefoot rabbitfish)	Intestinal contents	Pseudomonadota, Bacillota, Bacteroidota, Fusobacteriota, Mycoplasmatota, Cyanobacteriota	[39]
Various White Sea (arctic) fish	Posterior intestine	Pseudomonadota, Bacillota, Actinomycetota, Bacteroidota, Mycoplasmatota, Fusobacteriota	[40]
Various Mediterranean fish	Midgut	Pseudomonadota, Bacillota, Bacteroidota, Actinobacteriota, Patescibacteria, Fusobacteriota, Planctomycetota, and Dependentiae	[41]
Coastal fish of Hong Kong	Gastrointestinal contents	Pseudomonadota, Bacillota, Mycoplasmatota	[29]
Various deep-sea fish of Atlantic Ocean	Intestinal contents	Pseudomonadota, Bacteroidota, Bacillota, Actinomycetota, Ascomycota, Basidiomycota, Euryarchaeota, Spirochaetes	[30]
*Centroscyllium fabricii* (Black dogfish shark)	Gut contents	Actinomycetota, Pseudomonadota, Acidobacteriota (Acidobacteria), Bacillota, Chloroflexota	[42]
*Benthobatis moresbyi *(Dark Blind Ray)	Gut contents	Actinomycetota, Pseudomonadota, Acidobacteriota, Chloroflexota, Bacillota	[37]

Ref. = reference.

**Table 2:** Recent culture-independent studies of marine fish gut microbiome diversity. The most abundant/predominant taxa reported at family and genus level are given.

Fish	Sample	Abundant Genera	Abundant Families	Ref.
***Centroscyllium fabricii*** ** (Black dogfish shark)**	Gut contents	*Acinetobacter, Thalassobacillus,* *Alteromonas, Leeuwenhoekiella,* *Corynebacterium, Pseudonocardia,* *Pseudomonas*	NR	[42]
***Benthobatis*** ***moresbyi* (Dark Blind Ray)**	Gut contents	*Acinetobacter*	*Moraxellaceae*, *Koribacteraceae*, *Nitrospiraceae*	[37]
**White Sea (arctic) fish**	Posterior intestine	*Streptococcus*, *Sphingomonas*, *Micrococcus*, *Chthoniobacter*, *Pseudomonas*, *Corynebacterium*, *Staphylococcus*, *Acinetobacter*, *Propionibacterium*, *Vibrio*, *Photobacterium*, *Bacillus*	*Moraxellaceae*, *Vibrionaceae*, *Pseudomonadaceae*, *Propionibacteriaceae*, *Corynebacteriaceae, Micrococcaceae*	[40]
**Various Mediterranean fish**	Midgut	*Pseudoalteromonas,* *Bradyrhizobium, Diaphorobacter,* *Mycoplasma, Clostridium, Thaumasiovibrio, Microbulbifer*	*Xanthobacteraceae*, *Comamonadaceae*, *Pseudoalteromonadaceae*, *Clostridiaceae*, *Vibrionaceae*, *Propionibacteriaceae*, *Staphylococcaceae*, *Mycoplasmataceae*, *Flavobacteriaceae*, and *Peptostreptococcaceae*	[41]
**Various Antarctic fish**	Gastrointestinal contents	*Rhodococcus*, *Thermus*, *Acinetobacter*, *Propionibacterium*, *Streptococcus*, and *Mycoplasma*	NR	[32]
**Coastal fish of Hong Kong**	Gastrointestinal contents	*Clostridium*, *Photobacterium*, *Ralstonia*, *Acinetobacter*, *Thermus*, *Ralstonia*,	NR	[29]

NR = not reported, Ref. = reference.

**Table 3:** Bacteriocins, bacteriocin-like inhibitory substances and other AMPs identified from marine fish gut microbiome isolates. Many class IIa bacteriocins have been isolated from this microbial niche, covering a wide spectrum of susceptible organisms.

Molecule	Producer	Host, Source	Susceptible Organism(s)	Ref.
**Class I bacteriocins**				
Formicin	*Bacillus* *paralicheniformis* APC 1576	*Scomber scombrus*, intestine	*Clostridia* spp., *Bacillus* spp., *Listeria* spp., *Enterococcus* spp., *Streptococcus* *mutans*, *M. luteus*	[43]
Nisin Z	*Lactococcus lactis* subsp. *lactis*	*Paralichthys olivaceus,* intestine	*Streptococcus iniae*	[44]
Nisin Z	*Lactococcus lactis* TW34	*Odontesthes platensis*, intestine	*Lactococcus garvieae*	[45]
**Class IIa bacteriocins**				
Piscicocins Vla, Vlb	*Carnobacterium* *piscola* V1	salmon/trout, intestine	*Listeria* spp.	[46,47]
Divercin V41	*Carnobacterium* *divergens* V41	salmon or trout, intestine	*Carnobacterium piscicola*, *Listeria* spp.	[46,48]
Mundticin KS	*Enterococcus mundtii* Tw56	*Odontesthes platensis*, intestine	*Enterococcus* spp., *Listeria* spp., *M.* *luteus*, *Pseudomonas aeruginosa*, *Shewanella putrefaciens*	[49]
Enterocin R5	*Enterococcus* *lactis* RS5	*Sillago indica,* gut	*E. coli, S. enterica* Typhimurium, *S.* *aureus*, *P. aeruginosa. B. subtilis*, *B.* *cereus*, *Proteus vulgaris*	[50]
**Class Ild bacteriocins**				
CAMT6	*Enterococcus* *durans* YQ-6	*Larimichthys* *polyactis*, NR	*S. aureus*, *Bacillus* spp., *S.* *haemolyticus*, *P. acnes*, *Salmonella paratyphi*, *V*. *parahaemolyticus*, *P. foulis*, *E.* *aerogenes*, *Fusarium sylvaticum*, *Aspergillus fumigatus*	[51]
**Other AMPs /bacteriocin-like inhibitory substances**				
BaCf3	*Bacillus* *amyloliquefaciens* *BTSS3*	*Centroscyllium fabricii,* intestine	*Bacillus* spp., *Clostridium perfringens,* *Salmonella* Typhimurium, *Proteus vulgaris*	[52,53]

AMP = antimicrobial peptide; NR = not reported, Ref = reference.

## Data Availability

Not Applicable.
